# Comorbidities and outcomes among patients hospitalized with COVID-19 in Upper Egypt

**DOI:** 10.1186/s41983-022-00530-5

**Published:** 2022-08-12

**Authors:** Eman M. Khedr, Enas Daef, Aliae Mohamed-Hussein, Ehab F. Mostafa, Mohamed Zein, Sahar M. Hassany, Hanan Galal, Shimaa Abbas Hassan, Islam Galal, Amro A. Zarzour, Hebatallah M. Hassan, Mariam Taher Amin, Maiada K. Hashem, Khaled Osama, Ayman Gamea

**Affiliations:** 1 Department of Neurology and Psychiatry, Assiut University, Assiut, Egypt; 2 Department of Medical Microbiology and Immunology, Faculty of Medicine, Assiut University, Assiut, Egypt; 3 Department of Chest, Faculty of Medicine, Assiut University, Assiut, Egypt; 4 Department of Tropical Medicine and Gastroenterology, Alrajhi University Hospital, Assiut University, Assiut, Egypt; 5 Department of Internal Medicine, Assiut University, Assiut, Egypt; 6 Clinical Pathology Labs, General Chest Hospital, Assiut, Egypt; 7 Department of Anesthesia and Intensive Care, Assiut University, Assiut, Egypt; 8 Department of Chest, Faculty of Medicine, Aswan University, Aswan, Egypt; 9 Department of Public Health, Assiut University, Assiut, Egypt; 10 Department of Neurology and Psychiatry, South Valley University, Qena, Egypt; 11 Department of Neuropsychiatry, Faculty of Medicine, Assiut University Hospital, Assiut, Egypt; 12 Neuropsychiatric Department, Faculty of Medicine, Aswan University Hospital, Aswan, Egypt

**Keywords:** Comorbidities, COVID-19, Upper Egypt, Cardiovascular diseases, Neurological diseases, Chronic pulmonary diseases

## Abstract

**Background:**

The coronavirus disease 19 (COVID-19) pandemic has spread rapidly around the globe with considerable morbidity and mortality. Coexistence of comorbidities with COVID-19 had consistently been reported as risk factors for unfavorable outcome. We aimed to evaluate the impact of comorbidities in COVID-19 patients on the outcome and determine predictors of prolonged hospital stay, requisite for intensive care unit (ICU) admission. Four hundred and thirty-nine adult patients who are admitted through (June and July 2020) in our University Hospitals were included in the study. All participants were diagnosed with COVID-19 according to Egyptian Ministry of Health guidance as definite case or probable case.

**Results:**

Patients with comorbidities represented 61.7% of all cases. Constitutional symptoms especially myalgia and lower respiratory tract (LRT) symptoms such as dyspnea were significantly higher in patients with comorbidities (*P* < 0.05). Patients with comorbidities had significantly worse laboratory parameters. ICU admission was higher in patients with comorbidities (35.8%). Among different comorbidities 45.4% of cardiovascular diseases (CVD) cases were admitted in ICU followed by diabetes mellitus (DM) cases (40.8%). Also, patients with comorbidities needed invasive mechanical ventilation more than those without comorbidity (31 versus 10.7%, *P* < 0.001). Significant lower frequency of recovery was found in COVID-19 patients with comorbidities (59% versus 81%, *P* < 0.001) and death rate was significantly higher in cases with comorbidities (*P* < 0.001)***.*** The survival rates in cases with pre-existing CVD and neurological diseases were lower than those without disease (*P* < 0.002 and 0.001, respectively).

**Conclusions:**

Association of cardiovascular comorbid conditions including hypertension or neurological diseases including old cerebrovascular strokes together with COVID-19 infections carries higher risks of mortality. However, other comorbidities such as diabetes mellitus, chronic pulmonary or kidney diseases may also contribute to increased COVID-19 severity.

## Background

COVID-19 is a novel emerging, rapidly propagating illness that is overwhelming to most of resources of efficient health-care systems, and numerous hospitals, globally, are presently suffering a lack of ICU beds for critically ill COVID-19 pneumonic patients. In June 2020, a study published in SN Comprehensive Clinical Medicine investigated the belief that COVID-19 in a person with underlying health conditions or comorbidities "has an increasingly rapid and severe progression, often leading to death." The researchers looked at all the available data and found that having comorbidities also increases the chances of coronavirus infection [1].

Generally, one of the ultimate alarming clinical considerations is the presence of comorbidities. Comorbidities are associated with worse health outcome, more complex clinical management, and increased health-care cost. We should seek to provide the best local solutions in conjunction with the recent national guidelines to continue the proper management of patients while ensuring proper resource [2]. Chronic disorders participate in numerous topographies with communicable diseases, for instance the pro-inflammatory state, and the attenuation of the innate immune response which may possibly be allied etiologically to its pathogenesis [3]. Moreover, upregulation of angiotensin-converting enzyme in patients with chronic diseases are increasing the susceptibility to SARS-CoV-2 infection and the risk of disease aggravation [4].

A recent meta-analysis reported that underlying disease, including hypertension, diabetes mellitus, respiratory and cardiovascular disease [5], as well as obesity [6], may be risk factors for adverse outcomes. So further studies of comorbidities as a risk of fatality at different communities are required [7].

COVID-19-related mortality rate varies widely among counties [8]. To reduce the overall mortality rate, identifying risk factors associated with disease severity and poor outcome among COVID-19 patients is urgently needed.

Based on what we know at this time, adults of any age with previously mentioned conditions might be at an increased risk for severe illness from the virus that causes COVID-19 [1, 9]. The aim of this study is to evaluate the impact of diabetes, cardiovascular, chronic pulmonary, neurological, autoimmune, hepatic, renal and any other comorbidities or risk factors on COVID-19 patients outcome and determine predictors of prolonged hospital stay, requisite for ICU admission.

## Methods

This is a retrospective observational cohort study conducted in two major health-care centers in Egypt (8 specialized University hospitals). Both were designated to diagnose and treat moderate and severe cases of COVID-19.

All adult patients admitted during June and July 2020 were included in the study. Patients less than 18 years were excluded from the study.

All participants were diagnosed COVID-19 according to Egyptian Ministry of Health guidance. Evidence of SARS-CoV-2 infection was defined as Cases with definite COVID-19 if patients came with clinical symptoms of infection and polymerase chain reaction (PCR) of respiratory samples (nasopharyngeal) was positive.

Cases with probable COVID-19 if clinical symptoms of infection and chest CT was consistent with COVID-19 plus one or 2 laboratory investigations were positive) (lymphopenia, high serum ferritin and/or d-dimer), but PCR was negative or unavailable.

The samples of nasopharyngeal swab were stored at 2–8 °C up to 72 h.

RNA extraction of SARS-CoV-2 was done using (QIAamp Viral RNA Mini Kit Catalog number 52904 supplied by QIAGEN, Germany). Sample preparation using QIAcube instruments follows the same steps as the manual procedure (lyse, bind, wash and dilute).

Pathogen detection of SARS-CoV-2 RNA was done by (TaqMan™ 2019-nCoV Control Kit v1 (Catalog number A47532) supplied by QIAGEN, Germany) on the Applied Biosystem 7500 Fast RT PCR System, USA. It amplified and detected three viral genomic regions, reducing the risk of false negatives including the N protein (nucleocapsid gene), S protein (Spike gene), and open reading frame-1ab (ORF1ab) genes. Applied Biosystems™ TaqMan™ 2019-nCoV Control Kit v1 (Catalog number A47533) is a synthetic positive control that contains target sequences for each of the assays included in the TaqMan™ 2019-nCoV Assay Kit v1 (Catalog number A47532).

Clinical records and laboratory data were reviewed by the investigators in each study site and the following data were extracted for analysis: 

Demographic and clinical data: age, gender, presenting symptoms, comorbidities, and outcomes.

Laboratory investigations: complete blood picture, liver function tests, kidney function tests, d-dimer, serum ferritin and C-reactive protein (CRP).

Chest computed tomography (CT) findings was performed for each case.

Clinical outcomes: complete recovery, need for ICU admission, and death.

Classification of the patients: according to the presence or absence of pre-existing comorbidities which determined based on patients self-report on admission. The patients were classified into: Group I includes COVID-19 patients with at least one of the comorbidities (diabetes mellitus, hypertension, cardiac diseases, chronic pulmonary disease, chronic liver disease, chronic kidney disease, neurological disease (old cerebrovascular stroke, brain tumors, epilepsy, Parkinson disease dementia, multiple sclerosis, Parkinson`s disease, Alzheimer`s disease, myasthenia gravis and brain abscess. Autoimmune disease (systemic lupus erythematosus, rheumatoid arthritis, autoimmune vasculitis, Behcet’s disease, scleroderma. malignancy and any other disease or risk factor. Group II includes COVID-19 patients without any of comorbidities.

The research protocol was approved via the Ethical Review Committee of Faculty of Medicine before starting of the study (IRB no: 17300434)*.* Patients identifying information were concealed and each patient assigned for a code to insure privacy and confidentiality of the data.

Written informed consent was obtained from each patient or from first degree relatives.

### Statistical analysis

All statistical analyses were performed using IBM SPSS Statistics version 20 (SPSS Inc., Chicago, IL, USA). Categorical data were presented as frequencies and percentages, while Chi-square tests were used for comparisons between groups. Continuous data were reported as means ± standard deviations and tested for normality using the Shapiro–Wilk’s test. Where continuous data were normally distributed, the Student's T-test was used for comparisons between groups; where data were non-normally distributed, the Mann–Whitney test was used. For survival analysis, the outcome was tested related to time since the admission until death/end of the study period. For people who did not die, end of July 2020 was included as the final day of research; therefore, the study ended. The Kaplan–Meier survival curves were performed and differences in survival rates were analyzed by log-rank test. Univariate Cox regression was conducted to detect the hazard ratio (HR) of different comorbidities and variables with statistically different HR were included in a multivariate Cox regression model and adjusted for age and sex. In all statistical tests *P*-value < 0.05 was considered statistically significant.

## Results

**Fig. 1:**
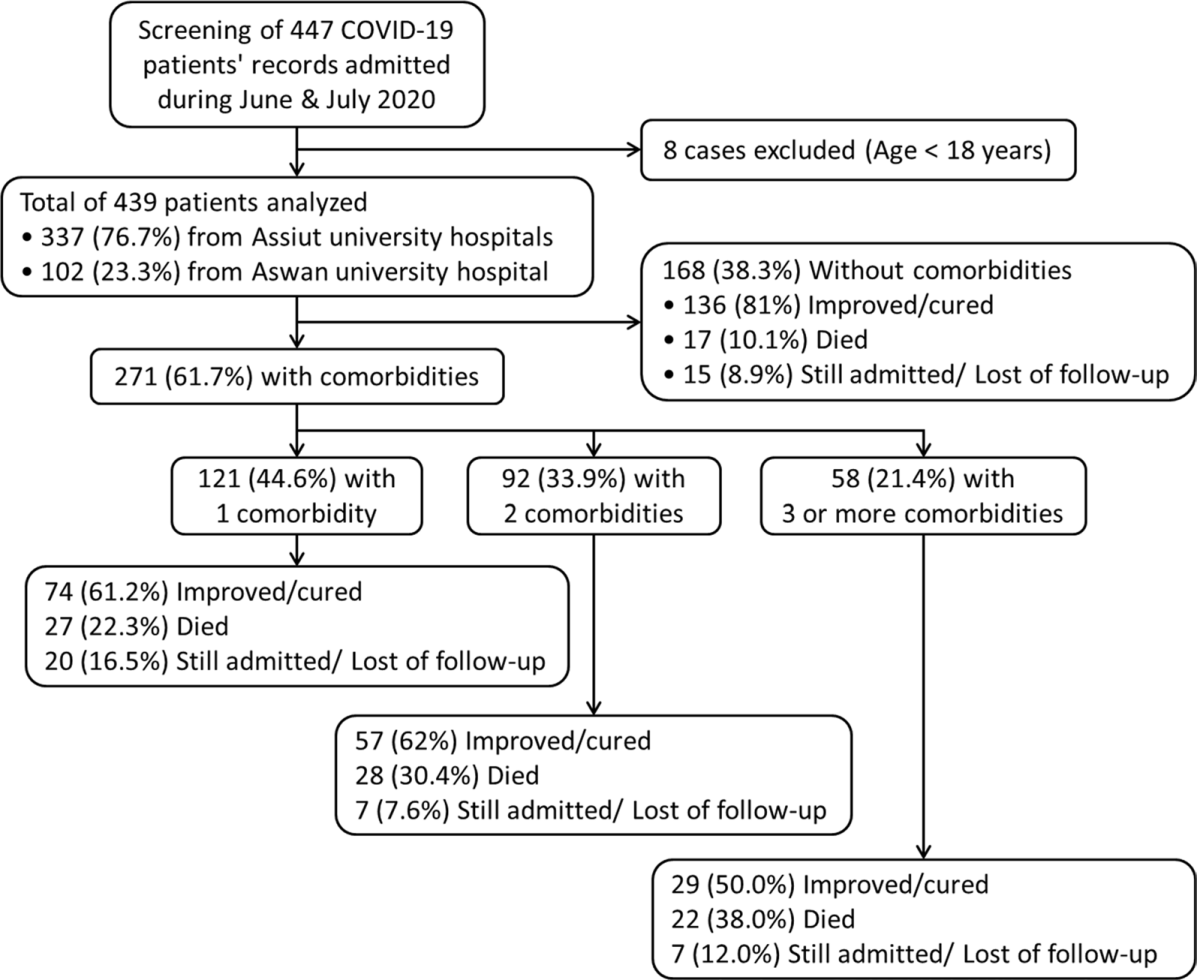
Flowchart of COVID-19 patients included in the study stratified according to number of comorbidities

Screening of 447 patients’ records was conducted and 8 cases were excluded due to their age (< 18 years old), then analysis of 439 cases was performed. Patients with comorbidities represented 61.7% of all cases as demonstrated in Fig. 1.

The mean age of studied patients was 51.2 ± 17.2 years old, and it was higher in patients with comorbidities (*P* < 0.001). Fever, and lower respiratory tract (LRT) symptoms especially dry cough were the most frequent symptoms (74.3% and 74.7%, respectively). Gastrointestinal tract (GIT) symptoms were present in 21.2% of patients; neurological symptoms in 43.8%. The most common presenting symptom was headache, dizziness and loss of smell and they were common in patients with comorbidities than patients without comorbidities. When comparing symptoms of the two groups, constitutional symptoms especially myalgia and LRT symptoms such as dyspnea were significantly higher in patients with comorbidities (*P* < 0.05). CT-chest showed bilateral ground glass opacities (GGO) in most of cases (82.6%) (Table 1).

**Table 1:** Demographic and clinical characteristics of COVID-19 patients included in the study (*n* = 439)

	All patients (*n* = 439)	Patients without comorbidity (*n* = 168)	Patients with comorbidities (*n* = 271)	*P*-value
Age (years)				
18–39	122 (27.8%)	96 (57.1%)	26 (9.6%)	< 0.001
40–59	147 (33.5%)	51 (30.4%)	96 (35.4%)	
60–79	153 (34.9%)	19 (11.3%)	134 (49.4%)	
≥ 80	17 (3.9%)	2 (1.2%)	15 (5.5%)	
Mean ± SD	51.2 ± 17.2	39.4 ± 15.0	58.5 ± 14.0	< 0.001
Gender, *n* (%)				
Male	224 (51%)	86 (51.2%)	138 (50.9%)	0.956
Female	215 (49%)	82 (48.8%)	133 (49.1%)	
Presenting symptoms, *n* (%)				
Fever	326 (74.3%)	118 (70.2%)	208 (76.8%)	0.129
Constitutional symptoms, *n* (%)	177 (40.3%)	55 (32.7%)	122 (45.0%)	0.011
Fatigue	125 (28.5%)	42 (25%)	83 (30.6%)	0.204
Bone pain	18 (4.1%)	7 (4.2%)	11 (4.1%)	0.956
Myalgia	63 (14.4%)	17 (10.1%)	46 (17%)	0.045
Anorexia	48 (10.9%)	12 (7.1%)	36 (13.3%)	0.051
URT symptoms, *n* (%)	110 (25.1%)	37 (22%)	73 (26.9%)	0.248
Sore throat	96 (21.9%)	31 (18.5%)	65 (24%)	0.173
Nasal congestion	24 (5.5%)	11 (6.5%)	13 (4.8%)	0.433
LRT symptoms, *n* (%)	328 (74.7%)	113 (67.3%)	215 (79.3%)	0.005
Cough	293 (66.7%)	105 (62.5%)	188 (69.4%)	0.137
Sputum	116 (26.4%)	24 (14.3%)	92 (33.9%)	< 0.001
Dyspnea	208 (47.4%)	51 (30.4%)	157 (57.9%)	< 0.001
GIT symptoms, *n* (%)	93 (21.2%)	36 (21.4%)	57 (21%)	0.922
Abdominal pain	11 (3.5%)	6 (3.6%)	5 (1.8%)	0.261
Nausea/vomiting	24 (5.5%)	6 (3.6%)	18 (6.6%)	0.169
Diarrhea	69 (15.7%)	31 (18.5%)	38 (14%)	0.215
Neurological symptoms, *n* (%)	192 (43.8%)	74 (44%)	118 (43.5%)	0.917
Headache	83 (18.9%)	27 (16.1%)	56 (20.7%)	0.232
Dizziness	63 (14.4%)	27 (16.1%)	36 (13.3%)	0.418
Vertigo	3 (0.7%)	1 (0.6%)	2 (0.7%)	0.860
Loss of smell	35 (8%)	15 (8.9%)	20 (7.4%)	0.560
Loss of taste	25 (5.7%)	10 (6%)	15 (5.5%)	0.854
CT-chest findings, *n* (%)				
Normal	16 (4.9%)	10 (9.2%)	6 (2.8%)	0.124
Bilateral GGO	270 (82.6%)	87 (79.8%)	183 (83.9%)	
Unilateral GGO	5 (1.5%)	2 (1.8%)	3 (1.4%)	
GGO and consolidations	36 (11%)	10 (9.2%)	26 (12%)	

URT: upper respiratory tract; LRT: lower respiratory tract; GIT: gastrointestinal tract; CT: computed tomography; GGO: ground glass opacities

The most frequent comorbidities in the studied group were cardiovascular diseases (69%) of patients with comorbidities followed by DM (54.2%) (Table 2).

**Table 2:** Frequency (number and percent) of different comorbidities among 439 COVID-19 patients

Comorbidity	Number	% from cases with comorbidities	% from all cases
Diabetes mellitus	147	54.2	33.5
Cardiovascular diseases	187	69.0	42.6
Hypertension	131 (70.0%)	63.4	39.2
Ischemic heart disease	8 (4.3%)	3.0	1.8
Hypertension and IHD	41 (21.9%)	15.1	9.3
AF	4 (2.1%)	1.5	0.91
Valvular disease	2 (1.1%)	0.7	0.46
Congenital cyanotic heart disease	1 (0.53%)	0.37	0.23
Chronic pulmonary diseases	31	11.4	7.1
COPD	12 (38.7%)	4.4	2.7
Asthma	10 (32.3%)	3.7	2.2
ILD	3 (9.7%)	1.1	0.68
SRBD	3 (9.7%)	1.1	0.68
Previous pulmonary embolism	2 (6.5%)	0.74	0.46
Pleural effusion	1 (3.2%)	0.37	0.23
Neuropsychiatric diseases	31	11.4	7.1
Old CVS	16 (51.6%)	5.9	3.6
Brain tumors	4 (12.9%)	1.5	0.91
Epilepsy	3 (9.7%)	1.1	0.68
Parkinson’s disease dementia (PDD)	2 (6.5%)	0.74	0.46
MS	2 (6.5%)	0.74	0.46
Parkinson’s disease (PD)	1 (3.2%)	0.37	0.23
Alzheimer’s dementia	1 (3.2%)	0.37	0.23
MG	1 (3.2%)	0.37	0.23
Brain abscess	1 (3.2%)	0.37	0.23
Kidney disease	21	7.7	4.8
Chronic kidney disease	18 (85.7%)	6.6	4.1
ESRF on RD	2 (9.5%)	0.74	0.46
Renal transplant	1 (4.8%)	0.37	0.23
Liver Disease	15	5.5	3.4
HCV	7 (46.7%)	2.6	1.6
HCV with cirrhosis	5 (33.3%)	1.8	1.1
Portal hypertension with bleeding varices	1 (6.7%)	0.37	0.23
Autoimmune hepatitis	1 (6.7%)	0.37	0.23
Liver transplant	1 (6.7%)	0.37	0.23
Metabolic and endocrinal diseases	10	3.7	2.3
Hypothyroidism	8 (80%)	3.0	1.8
Gout	2 (20%)	0.74	0.46
Autoimmune diseases	7	2.6	1.6
SLE	3 (42.9%)	1.1	0.68
RA	1 (14.3%)	0.37	0.23
Autoimmune vasculitis	1 (14.3%)	0.37	0.23
Behcet’s disease	1 (14.3%)	0.37	0.23
Scleroderma	1 (14.3%)	0.37	0.23
Other conditions/risk factors	9	3.3	2.1
Malignancy^a^	3 (18.8%)	1.1	0.68
Chronic bilateral LL ischemia	3 (18.8%)	1.1	0.68
Pregnancy	3 (18.8%)	1.1	0.68

^**a**^Acute leukemia, lymphoma and breast cancer on chemotherapy

IHD: ischemic heart disease; AF: atrial fibrillation; COPD: chronic obstructive pulmonary disease; ILD: interstitial lung diseases; SRBD: sleep related breathing disorders; ESRF on RD: end stage renal failure on regular dialysis; CVS: cerebrovascular stroke; HCV: hepatitis C virus; HCV: hepatitis C virus; CVS: cerebrovascular stoke; MS: multiple sclerosis; MG: myasthenia gravis; SLE: systemic lupus erythematosus; RA: rheumatoid arthritis

Patients with comorbidities had significantly worse laboratory parameters. Detailed data are included in Table 3*.*

**Table 3:** Laboratory data of COVID-19 patients included in the study

	Cases with available data	All patients	Patients without comorbidity	Patients with comorbidities	*P*-value
CBC					
Hemoglobin (g/dl)	393	12.2 ± 2.0	12.6 ± 1.9	11.9 ± 2.0	0.001
Anemic (HB < 12)		178 (45.3%)	54 (35.8%)	124 (51.2%)	0.003
WBCs (10^3^/ul)	405	9.2 ± 5.5	8.3 ± 4.8	9.8 ± 5.9	0.003
Low (< 4)		46 (11.4%)	23 (14.8%)	21 (9.2%)	0.014
High (> 10)		143 (35.3%)	42 (27.1%)	101 (40.4%)	
Neutrophil (%)	315	68.1 ± 20.4	63.5 ± 20.9	70.5 ± 19.7	0.013
Low (< 40)		26 (8.3%)	13 (11.8%)	13 (6.3%)	0.029
High (> 75)		144 (45.7%)	40 (36.4%)	104 (50.7%)	
Lymphocytes (%)	407	19.9 ± 14.6	23.0 ± 15.6	18.1 ± 13.7	0.001
Low (< 20)		242 (59.8%)	82 (53.6%)	160 (63.5%)	0.085
Lymphocytes (10^3^/ul)	407	1.4 ± 0.98	1.5 ± 0.99	1.4 ± 0.98	0.301
Low (< 1.5)		256 (62.9%)	92 (59.7%)	164 (64.8%)	0.542
Platelets (10^3^/ul)	391	275.2 ± 139.1	283.9 ± 118.7	269.8 ± 150.5	0.177
Low (< 140)		37 (9.5%)	4 (2.7%)	33 (13.7%)	< 0.001
High (> 450)		37 (9.5%)	10 (6.7%)	27 (11.2%)	
INR	256	1.1 ± 0.32	1.0 ± 0.12	1.1 ± 0.39	0.092
Ferritin (ng/ml)	262	749.0 ± 997.4	698.9 ± 1137.5	777.9 ± 909.0	0.002
High (> 291)		165 (63%)	50 (52.1%)	115 (69.3%)	0.005
d-dimer (mg/l)	297	2.5 ± 8.7	1.3 ± 3.4	3.5 ± 11.1	< 0.001
High (> 0.55)		180 (60.6%)	53 (41.7%)	127 (74.7%)	< 0.001
CRP (mg/dl)	289	57.8 ± 74.1	43.0 ± 74.5	68.3 ± 72.2	< 0.001
High (> 1)		282 (97.6%)	114 (95%)	168 (99.4%)	0.016
PCR					
Positive	416	354 (85.1%)	144 (88.9%)	210 (82.7%)	0.083
Negative		43 (10.6%)	18 (11.1%)	44 (17.3%)	

CBC: complete blood picture; WBCs: white blood cells; INR: international normalized ratio; PCR: polymerase chain reaction

**Fig. 2:**
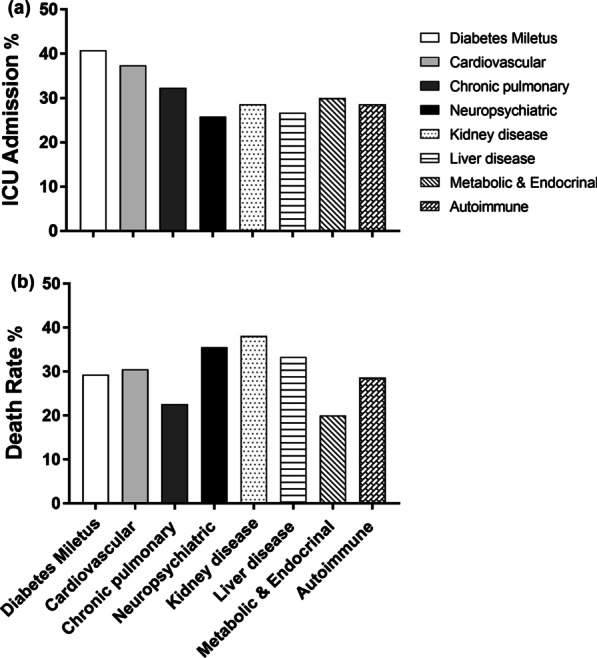
**a** ICU admission. **b** Death rate according to comorbidities

ICU admission was higher in patients with comorbidities as 35.8% of them needed ICU admission compared to only 16.4% of those without any comorbidity (*P* < 0.001). Among different comorbidities 45.4% of CVD cases were admitted in ICU followed by DM cases (40.8%) (Fig. 2a). Also, patients with comorbidities needed invasive mechanical ventilation more than those without comorbidity (31 versus. 10.7%, *P* < 0.001) (Table 4).

**Table 4:** Clinical outcomes of COVID-19 patients included in the study (*n* = 439)

	All patients (*n* = 439)	Patients without comorbidity (*n* = 168)	Patients with comorbidities (*n* = 271)	*P*-value
ICU admission	124 (34.1%)	28 (16.4%)	96 (35.8%)	< 0.001
Oxygen therapy				
No need	130 (29.6%)	76 (45.2%)	54 (19.9%)	< 0.001
Simple face mask	134 (30.5%)	53 (31.5%)	81 (29.9%)	
Venturi mask	45 (10.3%)	14 (8.3%)	31 (11.4%)	
Non-rebreathing mask	11 (2.5%)	3 (1.8%)	8 (3.0%)	
Noninvasive MV	17 (3.9%)	4 (2.4%)	13 (4.8%)	
Invasive MV	102 (23.2%)	18 (10.7%)	84 (31.0%)	
Outcome^a^				
Cured/improved	296 (67.4%)	136 (81%)	160 (59%)	< 0.001
Died	94 (21.4%)	17 (10.1%)	77 (28.4%)	
Duration of hospital stay	8.4 ± 6.1	8.5 ± 6.1	8.4 ± 6.0	0.853

ICU: intensive care unit

^**a**^49 patients (11.2%) were still admitted until the end of study period or lost follow-up

Recovery was recorded in nearly 68% of admitted COVID-19 cases (improved and discharged from hospital) with significant lower frequency of cure in patients with comorbidities (59% versus. 81%, *P* < 0.001) and death rate was significantly higher in cases with comorbidities (*P* < 0.001) (Table 4). Death rate in different comorbidity groups is illustrated in Fig. 2b.

The total number of patients needed invasive mechanical ventilation were 102 patients, the most frequent comorbidities associated with mechanically ventilated patients were diabetic patients, cardiovascular comorbidities including hypertension and cardiac diseases followed by chronic pulmonary diseases and chronic renal diseases. Patients with comorbidities needed mechanical ventilation more than patients without comorbidities (Table 5).

**Table 5:** Percentage needed mechanical ventilation from each comorbidity

	Number need MV* (MV/total cases)	% from comorbidity	% from MV (*n* = 102)
Comorbidity			
Diabetes mellitus	48/147	32.7	47.1
Hypertension	58/172	33.7	56.9
Cardiac diseases	20/56	35.7	19.6
Chronic pulmonary diseases	9/31	29.0	8.8
Neuropsychiatric diseases	10/31	32.3	9.8
Kidney disease	9/21	42.9	8.8
Liver disease	3/15	20.0	2.9
Metabolic and endocrinal diseases	3/10	30.0	2.9
Autoimmune diseases	2/7	28.6	2.0
Malignancy	1/3	33.3	0.98
Number of comorbidities			
No comorbidity	18/168	10.7	17.6
One comorbidity	31/121	25.6	30.4
Two comorbidities	31/92	33.7	30.4
Three comorbidities	19/49	38.8	18.6
Four comorbidities	3/9	33.3	2.9

**Fig. 3:**
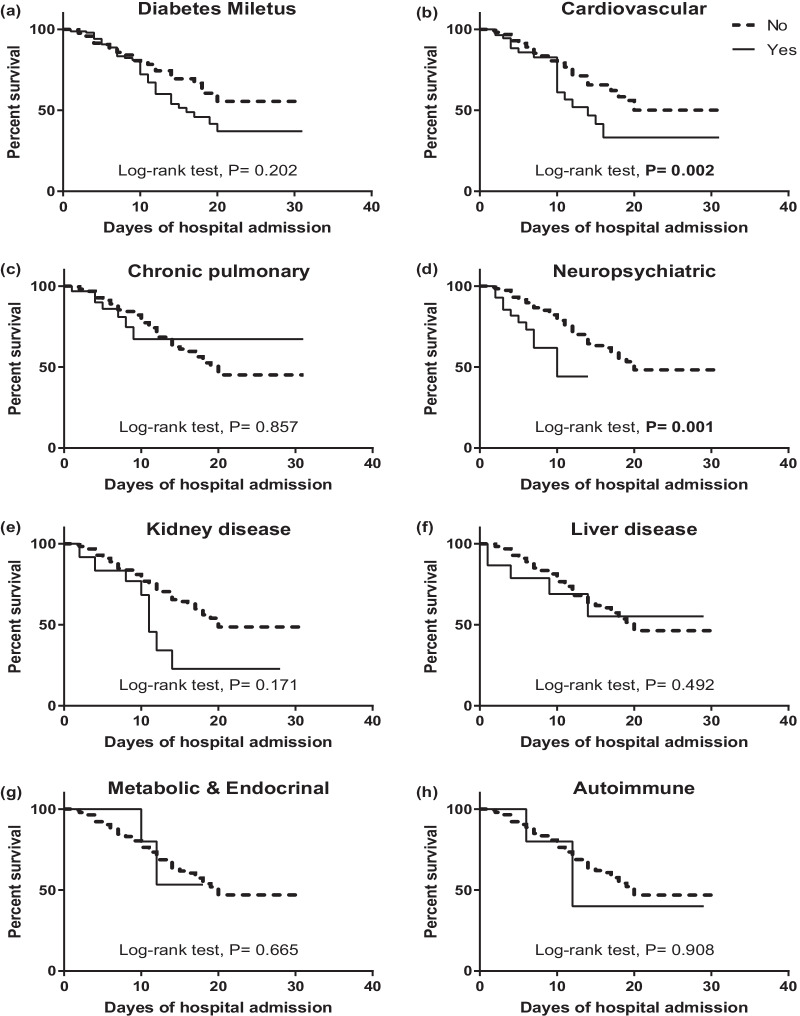
Kaplan–Meier survival curve of COVID-19 cases based on different associated comorbidities (*n* = 439)

**Fig. 4:**
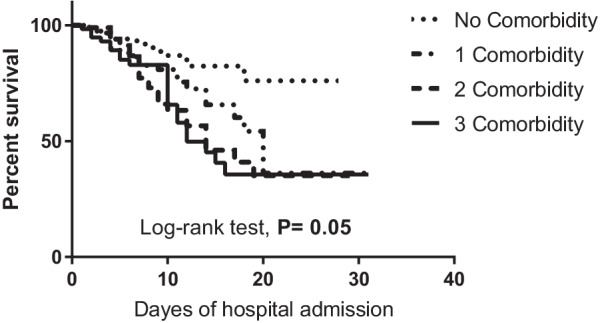
Kaplan–Meier survival curve of COVID-19 cases included in the study stratified according to number of comorbidities (*n* = 439)

Kaplan–Meier survival curves showed significant difference in survival rates in cases with pre-existing CVD and neurological diseases in which the survival rates are lower than those without disease (*P* < 0.002 and 0.001, respectively). Also, patients without any comorbidity showed higher survival rate than those with one, two or three or more comorbidities (74.2%, 34.5%, 34.3% and 35.5%, respectively) (Figs. 3 and 4).

In univariate Cox regression model, the older ages had higher risk of death than lower age groups as HR for 60–79 age group was 3.9 (95% confidence interval 2–7.8) and in cases > 80 years was 10.1 (95% CI 4–25.6). For different comorbidities, cases with CVDs and neurological diseases showed significant higher risk of death than others as HR in CVDs was 1.9 (95% CI 1.2–1.9) and in neurological diseases was 2.9 (95% CI 1.5–5.6). Also, number of comorbidities showed significant higher risk than those without any comorbidity as those with one comorbidity has a HR of 2 (95% CI 1.1–3.7) and with two comorbidities the HR as 2.6 (95% CI 1.4–4.7) while those with 3 or more comorbidities the HR as 2.9 (95% CI 1.5–5.6). After adjustment of significant factors in multivariate Cox regression, only higher age groups and neurological diseases showed higher risk for death (Table 6).

**Table 6:** Cox regression analysis of factors associated with overall survival in included COVID-19 cases in the study (*n* = 439)

Factors	Univariate analysis		Multivariate analysis	
	HR (95% CI)	*P*-value	HR (95% CI)	*P*-value
Age group (Ref. = 18–39)				
40–59	1.9 (0.89–3.8)	0.097	1.8 (0.81–3.9)	0.151
60–79	3.9 (2.0–7.8)	< 0.001**^**	3.7 (1.6–8.4)	0.002
> 80	10.1 (4.0–25.6)	< 0.001**^**	8.0 (2.7–24.0)	< 0.001
Gender (Ref. = Female)				
Male	1.2 (0.71–1.6)	0.737	–	–
DM	1.3 (0.86–1.9)	0.211	–	–
CVD	1.9 (1.2–2.8)	0.003^	0.81 (0.41–1.6)	0.542
Chronic pulmonary	0.93 (0.43–2.0)	0.859	–	–
Neuropsychiatric disease	2.9 (1.5–5.6)	0.002^	2.2 (1.1–4.7)	0.031
Kidney disease	1.7 (0.78–3.7)	0.184	–	–
Liver disease	1.4 (0.55–3.4)	0.502	–	–
Metabolic and endocrinal	0.74 (0.18–3.0)	0.672	–	–
Autoimmune disease	1.1 (0.27–4.4)	0.909	–	–
Number of comorbidities				
1	2.0 (1.1–3.7)	0.023**^**	1.3 (0.63–2.6)	0.496
2	2.6 (1.4–4.7)	0.002**^**	1.5 (0.61–3.5)	0.396
≥ 3	2.9 (1.5–5.6)	0.001**^**	1.3 (0.49–3.7)	0.564

CVD: cardiovascular diseases; DM: diabetes mellitus

## Discussion

Multiple comorbidities are associated with COVID-19 disease and may contribute to its progression or poor outcome.

In this study, as previously reported in reviews and metanalysis [1, 5, 10] cardiovascular diseases, including hypertension, (42.6%) and diabetes mellitus (33.5%) were the most common comorbidities associated with COVID-19 infection followed by chronic chest diseases and neuropsychiatric disorders (7.1%). This may be attributed to: (A) the older age of patients with comorbid diseases. Based on the current information, the elderly, a vulnerable population, with chronic health conditions such as diabetes and cardiovascular or lung disease not only a higher risk of COVID-19 infection [11], but also an increased risk of getting severe illness or even death if they become infected [12]. The weaknesses of advanced age are related to the function defense cells T and B, and to the excess production of type 2 cytokines, which can lead to a prolonged pro-inflammatory response, leading to unfortunate results [13]. (B) Common pathogenesis as chronic disorders collaborate several baseline topographies with communicable diseases, the pro-inflammatory status, and the attenuation of the innate immune response. Diabetes occurs in part because the accumulation of triggered innate immune cells leads to the release of inflammatory markers, principally IL-1β and tumor necrosis factor α, that promote generalized resistance to insulin and destruction of β-cell [14]. (C) Immunity depletion by impairing macrophage and lymphocyte function which may make individuals more susceptible to infectious diseases and its complications [3]. (D) Up-regulation of angiotensin-converting enzyme 2 (ACE2) genes expression in different parts of the body, such as heart and lungs, in patients with diabetes, or CVD, increasing the susceptibility to SARS-CoV-2 infection and the risk of disease aggravation as it has been identified as an important functional receptor for SARS-CoV-2 invasion [4, 15].

Cardiovascular comorbid conditions are associated with a variety of the worse outcomes for COVID-19 in literatures [16–18]. Moreover, association between hypertension and other coexisting comorbidities, such as HIV, kidney, or other cardiovascular diseases raises the hazard rate more than the other comorbidities [18]. In this study, 37.4% of patients with CVDs including hypertension admitted to ICU and 30.5% of them were deceased. Li and colleagues [17] found that the levels of inflammation indicators like CRP, serum ferritin and ESR were increased in COVID-19 patients and associated with the severity of the disease. Furthermore, the levels of these indicators in the patients with CVD were ominously higher than those without CVD, which indicate that COVID-19 cases with CVD had additional potential to practice an inflammatory storm, which ultimately clues to prompt worsening of these patients’ conditions. Through the course of contagion, inflammation of the lung tissue inhibits the exchange of oxygen in the alveoli, progressing to generalized tissue hypoxia, which triggers the fibrinolytic system. Moreover, compared with the non-CVD group, the ranks of d-dimer and fibrinogen levels were greater among CVD group, which indicate that they were more susceptible to hyper-coagulability. A hyper-coagulable state raises the hazard of pulmonary embolism, which can explain the sudden occurrence of complications such as hypoxia and heart failure [17]. All those results were harmonized with the current study which displayed significantly higher levels of all inflammatory markers as well as d-dimer level in patients with comorbidities. However, due to the overlap between different conditions (33.9% of patients had 2 comorbid conditions and 21.4% had three or more), we could not evaluate patients with CVD alone.

Unlike most of the published literatures, even a recent observational study carried out in the same geographical area which stated that “pre-existing DM may predict unfortunate 30 days in hospital outcome” [19], diabetes mellitus was not found to be a risk factor for death in patients with COVID-19 (HR 1.3; 95% CI 0.86–1.9; *P* < 0.211) despite that 40.8% of patient with DM were admitted to ICU meanwhile, 29.3% were deceased. In a cohort study of 7337 patients with COVID-19, it was shown that those with type 2 diabetes not only prerequisite augmented interferences for their stay in hospital versus those that were non-diabetic, but also had an increased mortality rate [20]. In the current study, in contrast with the above-mentioned ones, we considered DM as a comorbidity when it is self-reported by patient (previous diagnosis and regular treatment) not on the baseline random blood glucose at admission. According to Yan and colleagues, non-survivors with diabetes had higher levels of leukocyte count, neutrophil count, C-reactive protein, pro-calcitonin, ferritin, receptors of interleukin-2, interleukin-6, TNF-α, and lower lymphocytic count than survivors, which indicated diabetic non-survivors had more sever inflammatory response [21].

Despite the notable COVID-19 cases presentation with acute cerebrovascular accidents [22], pre-existing neurological conditions with their wide spectrum of diseases also related to COVID-19 infection and mortality. One survival analysis in 2070 Brazilian patients found that a risk of 3.9 times in people with neurologic disease (95% CI 1.9–7.8; *P* < 0.001) [4]. The increased severity of COVID-19 among cases with cerebrovascular illness may be attributed to coexistence of cerebrovascular diseases with other risk factors such as older age, cardiovascular diseases, and diabetes mellitus. Besides, the existence of brain medullary cardiorespiratory or autonomic nervous system dysfunction precipitates blood pressure fluctuation and dysfunction in the respiratory system increasing the hazard of acquiring opportunistic infections. Moreover, the relative immobility in post-stroke patients, increases the risk for hyper-coagulable state [23].

Among other comorbidities, chronic pulmonary diseases including obstructive pulmonary disease (COPD) and moderate-to-severe bronchial asthma are at higher risk of sever COVID-19 progression since this virus affects their respiratory tracts, leading to increased bronchospasm, pneumonia, and acute respiratory distress [12]. Asthma may not be a risk factor because of reduced angiotensin-converting enzyme-2 (ACE2) gene expression in airway cells of asthma patients that would be expected to decrease the severity of SARS-CoV-2 infection which uses ACE2 as its cellular receptor [24]. We suggest that the degree of asthma control, medications used for asthma control, difference in patient ages and the coexisting conditions may explain this disagreement. Besides, a high-quality cohort study with longer time frame and larger number of patients is needed to explain asthma COVID-19 interaction.

Unfortunately, a smaller number of patients with other comorbidities such as chronic kidney disease (CKD), liver, metabolic, endocrine, and autoimmune diseases as well as patients with malignancy were included in this study. However, the highest death rate among all the comorbid conditions in this study was (38.1%) of patients with kidney diseases. Cases with liver disorders also had relatively high mortality rate (33.3%). In literature, the rate of mortality in COVID-19 patients with CKD and Liver diseases was found to be (53.33%) and (17.65%), respectively [22]. Chronic kidney diseases are associated with dysregulated inflammation, immune system, and levels of ACE2 receptors in kidneys which may explain the severity and mortality due to COVID-19 in patients with CKD [22]. It is worth noting that in this study patients with comorbidities had significantly higher renal function tests together with elevated inflammatory biomarkers.

The limitations of the current study include the following: (a) the retrospective design of the study with the heterogeneity of data recoding in different hospital sectors; (b) the changing treatment protocol and admission policy during the study period; (c) some subjects having more than one underlying comorbidity.

## Conclusions

Coexistence of cardiovascular comorbid conditions including hypertension or neurological diseases together with COVID-19 infections carries higher risks of mortality. However, other comorbidities such as diabetes mellitus, chronic pulmonary or kidney diseases may also contribute to increased COVID-19 severity. Special attention should be taken during dealing with such cases. Future studies with larger number of patients in wider varieties of comorbidities and longer duration evaluating their impact on survival are desired.

## Data Availability

The datasets used and/or analyzed during the current study are available from the corresponding author on reasonable request.

## Abbreviations

ACE2: Angiotensin-converting enzyme 2
CKD: Chronic kidney disease
COPD: Chronic obstructive pulmonary disease
COVID-19: Coronavirus disease 19
CI: Confidence interval
CRP: C-reactive protein
CVD: Gastrointestinal diseases
DM: Diabetes mellitus
GGO: Ground glass opacities
GIT: Gastrointestinal tract
HR: Hazard ratio
ICU: Intensive care unit
LRT: Lower respiratory tract
PCR: Polymerase chain reaction
